# Participatory Intervention Development of a Peer-Guided Self-Help App for Anxiety Disorders: Mixed Methods Study

**DOI:** 10.2196/62781

**Published:** 2025-06-20

**Authors:** Laura Duddeck, Timo Stolz, Christian Zottl, Thomas Berger, Johanna Boettcher

**Affiliations:** 1 Department of Psychology and Psychotherapy Psychologische Hochschule Berlin Berlin Germany; 2 Department of Education and Psychology Freie Universität Berlin Berlin Germany; 3 Nullachtvierzehn Unternehmergesellschaft (Haftungsbeschränkt) Berlin Germany; 4 Deutsche-Angsthilfe Eingetragener Verein Munich Germany; 5 Department of Psychology and Psychotherapy University of Bern Bern Switzerland

**Keywords:** peer-guided self-help, participatory design, integrate, design, assess, and share framework, digital mental health intervention, anxiety self-management, user engagement, qualitative usability study

## Abstract

**Background:**

Anxiety disorders affect approximately 27% of the global population, posing a major mental health challenge. Limited access to treatment due to resource constraints highlights the need for scalable solutions. Web-based self-help programs provide low-threshold access to evidence-based strategies. When guided by peers, these programs enhance engagement and acceptability by merging autonomy with support. Peer-guided self-help apps offer a cost-effective alternative to traditional care, reaching those who might otherwise remain untreated.

**Objective:**

This study aims to describe the development of a peer-guided self-help app for anxiety, incorporating input from individuals with lived experience. It assesses user feedback on usability and helpfulness during the development process.

**Methods:**

The intervention was developed in 3 iterative stages using the integrate, design, assess, and share framework. In stage 1, a prototype was cocreated by employees of a German self-help organization with lived experience, software engineers, and psychologists. In stage 2, qualitative feedback was collected from a focus group (n=5) and interviews (n=4), with participants recruited through group leaders of the organization. The research team directly contacted the participants. Qualitative data were analyzed with inductive and deductive content analysis (interrater reliability Cohen κ=0.88), which informed the minimum viable product (MVP) development. In stage 3, the MVP was pilot-tested with a larger online sample (N=126) recruited via the organization’s website, accessible to all. Anxiety (Generalized Anxiety Disorder-7) and well-being (the World Health Organization-Five Well-Being Index) were assessed at baseline, 4, 8, and 12 weeks. Use metrics (eg, log-ins, time spent, and feature use) were recorded automatically. Quantitative data were analyzed descriptively.

**Results:**

Stage 1 produced no data. In stage 2, feedback revealed unclear functionality, confusion in peer interaction, and safety concerns, leading to MVP revisions. In stage 3 (N=126), engagement was low—average log-ins were 3.15 (SD 14.37), with only 20 (SD 15.9) participants completing follow-ups. While many joined exposure (79/126, 62.7%) or activity scheduling groups (104/126, 82.5%), 123 (98.4%) did not send messages, undermining peer support goals. Baseline scores showed moderate anxiety (Generalized Anxiety Disorder-7: mean 10.52, SD 5.15), low well-being (World Health Organization-Five Well-Being Index: mean 15.80, SD 6.17), and low social support (Oslo Social Support Scale-3: mean 7.25, SD 2.68), consistent with the target group. Low engagement and high attrition indicated usability problems and limited perceived value.

**Conclusions:**

Despite rapid sign-ups, user engagement was low and dropout rates high, indicating poor acceptance. Key barriers included user confusion, underused peer features, and technical issues. Future development should include structured onboarding for better clarity. Peer engagement be improved with prompts and enhanced safety perception. The participatory approach was challenging and fell short of expectations. Smaller testing phases with regular user feedback will ensure user-centered refinement. Insights from successful peer communities can inform a more intuitive, engaging design.

## Introduction

### Background

Anxiety disorders are among the most prevalent mental health conditions globally, representing 26.9% of all mental health issues worldwide [1], putting considerable strain on mental health services. In Germany, individuals seeking psychotherapy frequently face waiting periods of 3 to 9 months [2]. These delays are linked to negative impacts on individual well-being and rising health care costs [3]. Given these challenges, scalable and accessible intervention formats are receiving more attention in mental health research.

One approach explored in this context is self-help, which supports individuals in managing their mental health challenges independently and may reduce reliance on professional care [4]. Earlier studies indicate a general preference for self-help among certain populations [5]. More recent findings support this trend; for instance, Samuel and Kamenetsky [6] found that students were more likely to seek help from friends, family, or the internet than the university’s health and counseling center.

With internet-based interventions (IBIs) growing in availability over the past 2 decades, self-help has become increasingly accessible. IBIs have demonstrated effectiveness across various mental health conditions, particularly anxiety disorders [7], and have been considered a potentially suitable option for individuals who are hesitant to access traditional mental health services [8]. These interventions are commonly based on cognitive behavioral therapy and vary in their level of support; *guided interventions* offer (professional) support throughout the treatment process, while *unguided interventions* rely entirely on self-directed engagement, independent of human support. Although meta-analyses often show advantages of guided programs (eg, improved outcomes [9] and adherence [10]), randomized controlled trials (RCTs) do not consistently confirm superior outcomes for guided interventions (eg, concerning anxiety [11-13] and depression [14,15]). Different forms of guidance have been studied, including nonprofessional guidance, technical support, or diagnostic interviews [16-20], with promising results.

These findings have led to increased interest in how digital interventions might be structured to support adherence and outcomes in the absence of professional guidance. One potential form of guidance is peer support. Peer support as a key concept in traditional self-help refers to mutual support between individuals who share experiences in overcoming common challenges [21,22]. According to this definition, peers offer relatable guidance and emotional and motivational support and serve as role models for coping behaviors [23,24]. Peer support offers a balanced approach between fully guided and completely unguided interventions. It can strengthen adherence [25] and social support [18] and help address common barriers to the uptake of IBIs, such as comprehension issues, lack of time, or insufficient personalization [26,27]. In line with social identity theory [28], peer support can activate a sense of belonging and cohesion and reduce mental health stigma. It also provides an opportunity for social learning [29], with peers demonstrating adaptive coping strategies and providing behavioral models for users to emulate.

Despite increasing evidence for the efficacy of IBIs in treating anxiety, little is known about how peer-guided online formats can enhance engagement and outcomes. Most existing IBIs rely on professional guidance or are entirely self-guided, leading to limited research on intermediate models involving peers with lived experience. A recent study addressing this gap examined an online written exposure therapy program designed for veterans with posttraumatic stress disorder. Supported by peer coaches, the program demonstrated promising improvements in symptoms [30]. While the intervention successfully decreased posttraumatic stress disorder and depressive symptoms, it also revealed substantial challenges related to engagement and dropout rates. Over 50% of the participants did not start the program, and nearly half of those who did ultimately dropped out. These results underscore the need for careful design in digital mental health interventions, specifically targeting technical barriers and improving user engagement.

Peer support may benefit from being integrated into interventions that align with the needs and expectations of target users to enhance relevance and usability. Participatory intervention development (PD) offers such a pathway by actively involving end users in the design process. Instead of positioning users as passive recipients, PD encourages cocreation and continuous feedback to improve acceptance and effectiveness [25,31-33]. A recent study by Gonsalves et al [34] examines the co-design of “Baatcheet,” a peer-supported, web-based storytelling intervention aimed at young individuals facing mental health issues in India. The project used iterative co-design workshops to develop a culturally relevant platform that integrates user-generated stories, reflection activities, and peer support, aiming to reduce stigma and foster connections. This research highlights the significance of incorporating peer components and using participatory design to enhance the authenticity, relevance, and engagement of digital interventions, providing valuable insights for similar app-based initiatives. However, despite its advantages, PD also entails challenges, such as recruiting representative participants [35], managing stakeholder conflicts [36], and time-intensive processes in gathering and analyzing user feedback [37]. Therefore, the evidence from PD regarding the challenges of PD’s impact on intervention efficacy is limited due to insufficient outcome data and inconsistent results across specific contexts [32,35,38]. Nevertheless, interventions seem to benefit from PD, as demonstrated in various contexts, including somatic complaints, youth mental health, and outpatient psychotherapy. Examples include a pharmacy system intervention involving pharmacy staff and older adults [37], a mental health e-Clinic developed in collaboration with young people and professionals [33], and a blended outpatient psychotherapy program incorporating input from psychotherapists and individuals with lived experience [39]. In addition, the value of PD extends beyond developing a final product. Participating in the PD process is associated with increased knowledge, enhanced mental health literacy, and opportunities for peer exchange [35]. So far, no study has applied a participatory approach to the development of a peer-guided self-help app for anxiety.

### Objectives

This study addresses this gap by (1) developing a peer-guided self-help app for anxiety through a participatory approach using the integrate, design, assess, and share (IDEAS) framework and (2) evaluating its uptake, adherence, and preliminary effects on anxiety and well-being. The aim is to explore the potential of peer support in digital self-help and contribute to more user-centered, scalable mental health care solutions.

## Methods

### Overview

This study is based on the recurring phases 1 to 8 of the IDEAS framework [31], which served as a flexible orientation rather than a rigid sequence: (1) empathize with target users; (2) specify target behavior; (3) ground in behavioral theory; (4) ideate creative implementation strategies; (5) prototype potential products; (6) gather user feedback; (7) build a minimum viable product (MVP), defined as the first fully functioning version of the program that includes all core features; and (8) pilot-test.

### Intervention Development With Individuals With Lived Experience (Phases 1-7)

#### Participants

The development process began in August 2020 with a team of 3 individuals who were affected by anxiety disorders and were also active members of a German self-help association. This association aimed to create a self-help app and secured funding for the project in collaboration with 2 software development company employees, including a programmer (TS) and a project manager. The funding enabled the initiation of the development process. After 19 months, 2 psychologists (JB and LD) joined the team. The participants in these phases were not specifically recruited. They applied collaboratively for further funding to support the app’s development. The selection criteria for the software company included previous experience in developing health apps, while the psychologists were required to have expertise in online interventions.

#### Procedure

Weekly meetings involving all team members were convened to discuss target behaviors, core interventions, implementation strategies, linguistic expression, usability, and app design. Software engineers commenced programming while representatives from the self-help organization offered feedback on the technical prototypes. Psychologists contributed clinical insights and developed a research plan for the systematic piloting of the app. All team members conducted systematic testing of the prototype, adhering to a predefined testing schema.

#### Analysis

No data were systematically evaluated or analyzed.

### Testing the Prototype: Focus Group and Individual Interviews (Phases 6 and 7)

By September 2022, a prototype incorporating fundamental functionalities had been prepared for the initial phase of external user testing. The objective was to collect external feedback (phase 6) and subsequently enhance the prototype into an MVP (phase 7).

#### Participants

Leaders of face-to-face self-help groups were informed about the study by the organizing committee of the self-help organization. They were asked to either participate themselves or invite their self-help group members to join. Detailed information was sent to those who expressed interest via email (n=9), including study information, the app link, and a date for the online focus group. For those who were unable to attend the focus group, individual interviews were arranged. While the sample size (n=9) may appear modest, it is consistent with qualitative research standards for early-stage, exploratory studies aimed at identifying key themes and informing intervention design. According to Guest et al [40], thematic saturation often occurs within the first 6 to 12 interviews, especially when the research question is narrow and the participant group is relatively homogeneous. In this study, the combination of a focus group and individual interviews provided complementary perspectives. Core themes were consistently observed across both formats, indicating that the most salient user needs, preferences, and barriers were captured. No specific inclusion or exclusion criteria were defined; the only requirement was that participants had to be members of self-help groups within the German self-help organization.

#### Measures

A focus group (n=5) and individual interviews (n=4) were conducted, moderated by LD (a PhD student with advanced training in psychotherapy) and Pauline Becker (an academic assistant). The video-based focus group lasted approximately 90 minutes, while each individual interview lasted around 60 minutes. Participants in the focus groups and individual interviews were asked to share their expectations regarding the app’s effects and content (eg, “What did you expect from the app when you started using it?”) and identify the strengths and weaknesses of the app, focusing on its general effects and benefits (eg, “What do you think are the app’s biggest strengths and weaknesses?”). In addition, participants were encouraged to share their experiences regarding communication with other test users (eg, “How did you experience the exchange with others in the app?”). Finally, they were asked to describe the barriers and facilitators they encountered in the different modules (eg, “What was difficult or disruptive? What keeps you from using this feature?”). Both the focus group and the individual interviews used a semistructured interview format (Multimedia Appendices 1 and 2). The format allowed for deviations based on participants’ perspectives, opinions, and experiences. Topics discussed in the focus group were documented in an online tool, which was visible to all group members during the interview and shared afterward so that participants could provide additional feedback and comment on any misunderstandings or missing aspects.

#### Qualitative Analysis and Interrater Reliability

The focus group and interview recordings were anonymized and transcribed based on semantic content transcription guidelines by Dresing and Pehl [41], using MAXQDA (version 20; VERBI Software). Transcriptions were largely verbatim, with adaptations to standard written German where appropriate. Dialects were standardized, syntactic errors were retained, stuttering was smoothed, incomplete sentences were marked with interruption symbols, and paralinguistic elements (eg, laughter, pauses, and emphasis) were systematically noted. Each speaker’s turn, including brief interjections, was formatted into its paragraph. Participants and interviewers were pseudonymized using numerical codes.

The qualitative analysis followed a structured, multistep coding process integrating both deductive and inductive approaches. First, level 1 categories were developed deductively from the interview guide (Multimedia Appendices 1 and 2), aligning with overarching research aims (eg, expectations, interaction, modules, which encompassed activity scheduling, exposure, and psychoeducation), user experience, and functionality. Two raters independently analyzed the same 27% of the data using these preliminary categories. Discrepancies in coding were resolved through consensus discussion between the raters, using specific excerpts to refine category definitions and improve clarity. Thereafter, the 2 raters developed level 2 categories inductively from the material (eg, community, interpersonal exchange, suggestions for improvement, and system bugs). Again, discrepancies in coding were resolved through consensus discussion. The process was rehearsed for level 3 categories (eg, time management, direct messaging, and upload features). No third party was required. Once sufficient category coverage and definition precision were achieved, the finalized framework was applied to the full dataset by both raters (Multimedia Appendix 3). To ensure consistency, interrater reliability was calculated using MAXQDA 2022. The degree of code overlaps across various text segments served as the reliability criterion. Interrater reliability was quantified using the Cohen κ statistic, following the method established by Brennan and Prediger [42]. The resulting Cohen κ value of 0.88 indicated a high level of agreement among the independent raters. Although member checking was not conducted, the refinement of categories was guided by direct alignment with participant expressions, thereby supporting interpretative validity.

#### Additional Testing

The app was tested by a group of psychology students (n=9) to evaluate its user-friendliness in greater detail. They used the app for 3 weeks. Usability feedback was collected during 2 face-to-face group discussions led by the psychologists (JB and LD) and the software developer (TS). This feedback was incorporated into the development of the MVP but was not further analyzed.

### Testing the MVP: Questionnaires and Log Data (Phase 8)

#### Overview

Using feedback from the focus groups and interviews, the prototype was improved to develop an MVP. This version was tested with a larger group of users to evaluate its acceptability and gain an initial impression of the intervention’s potential effectiveness. The pilot trial was preregistered (DRKS00030781).

#### Participants

Test users were recruited from the website of a German self-help association. The study website offered comprehensive details about the app, its content, and the study’s objectives and procedures. Interested individuals could register using the link to the app. No inclusion or exclusion criteria were applied; any interested individuals could register for the app, regardless of symptom severity or membership in the German self-help association.

#### Measures

##### Quantitative Measures

Quantitative data were collected using validated self-report instruments at 4 time points: baseline time point (T0), 4-week time point (T1), 8-week time point (T2), and 12-week time point (T3). These assessments aimed to capture changes in anxiety, well-being, life satisfaction, self-efficacy, and social support, which were core constructs aligned with the app’s objective to reduce anxiety symptoms and promote psychological empowerment through peer-guided self-help. Participants received automated email reminders to complete questionnaires at various time points. Demographic information was collected at T0 to describe the sample composition. Anxiety symptoms were assessed using the Generalized Anxiety Disorder-7 (GAD-7) [43], a widely used 7-item measure specifically developed for screening and monitoring generalized anxiety in clinical and community samples over the previous 2 weeks (eg, “nervousness, anxiety, or tension”). It was chosen for its brevity, high sensitivity to change, and excellent internal consistency (Cronbach α=0.85) [44]. GAD-7 aligned directly with the app’s primary goal of anxiety reduction. Well-being was measured using the World Health Organization-Five Well-Being Index (WHO-5) [45], a 5-item scale assessing positive mood, vitality, and general interest in daily life (eg, “I have felt calm and relaxed”). It complemented the GAD-7 by focusing on positive indicators of psychological health, relevant for tracking subjective improvement during self-help interventions. The WHO-5 has shown high internal consistency (Cronbach α>0.92) [46]. Life satisfaction was captured via the General Life Satisfaction Short Scale-1 (L-1) [47], a single-item measure validated as a global index of subjective well-being (“How satisfied are you overall with your life at the moment?”). Its inclusion allowed efficient assessment of broader quality-of-life outcomes that may improve through symptom reduction and increased social connectedness. The L-1 shows good retest reliability (*r*=0.67-0.70) [47] and is especially suited for low-burden measurement in digital interventions. General self-efficacy was measured using the Short Scale for General Self-efficacy Beliefs (ASKU) [48], a 3-item instrument evaluating perceived coping ability. It reflects users’ belief in their capacity to manage challenges (eg, “In difficult situations, I can rely on my abilities.”). The ASKU demonstrates good internal consistency (McDonald ω=0.81-0.86) [48] and construct validity in both clinical and nonclinical samples. To assess mental health-specific self-efficacy, the Mental Health Self-Efficacy Scale (MHSE) [49] was included. This 6-item measure evaluates confidence in handling emotional distress over the next month (eg, “How confident are you that on an average day over the next month...you will be able to effectively handle stress, anxiety, and depression?”) and was specifically chosen for its relevance to expected app outcomes, including anxiety and stress management. The MHSE has shown high internal consistency (Cronbach α=0.89) [49]. Perceived social support was evaluated using the Oslo Social Support Scale [50], a brief 3-item measure assessing structural and functional support (eg, “How many people are close enough for you to count on during serious personal problems?”). As peer connection was central to the intervention, this measure determined whether app use enhanced users’ support perception. Despite moderate internal consistency (Cronbach α=0.64) [50], it has been widely used in population-based studies due to its brevity and adequate construct validity.

##### Log Data

The log data delineated the use behavior pattern of each user. The app automatically recorded various use metrics. Our examination encompassed the number of visits to the app, the volume of messages sent, the average number of page hits per visit, the frequency of visits over time, the duration of active engagement within different modules, and whether users participated in or initiated exposure or behavioral activation groups (refer to the description of the app in the subsequent sections).

#### Analysis

The questionnaire and log data underwent descriptive analysis, which involved calculating means and SDs using SPSS (version 29; IBM Corp). This analysis provided insights into the characteristics of the study sample and app use. The log data captured participants’ adherence and engagement with the app, thereby offering insight into the app’s acceptability. Because only a very small proportion of individuals completed the questionnaires (refer to the subsequent sections), we forewent applying inferential statistics.

#### Deviations From the Trial Registration

**Figure 1:**
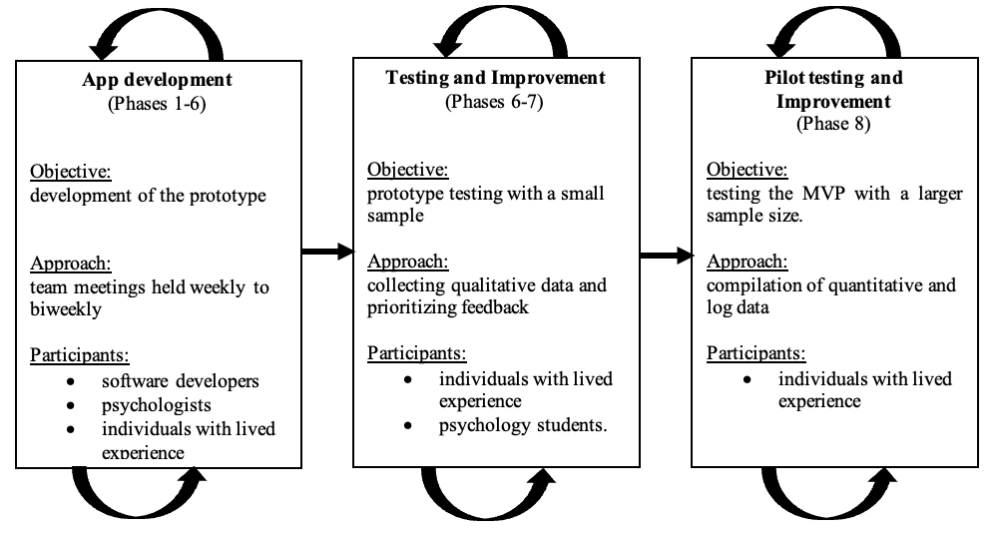
Overview of the development process for a self-help app addressing anxiety, based on the integrate, design, assess, and share framework. MVP: minimum viable product.

Due to low user engagement with the app, data collection ceased at T3. No follow-up measurements were conducted at the 6-month time point, and no client change interviews occurred at the 8-week time point. The planned analyses were limited to a descriptive evaluation in SPSS (Figure 1).

### Ethical Considerations

#### Overview

This study received approval from the research ethics committee of the Psychologische Hochschule Berlin (EK202206). Informed consent outlined the study’s objectives and procedures, potential benefits, and risks for participants and included data protection information, such as pseudonymization, anonymization, data processing, and the right to withdraw consent at any time.

#### Informed Consent in Prototype Development (Phases 1-6)

No explicit informed consent was obtained from individuals during this phase of the study, as no external participants were involved in the collaborative intervention development; no data were systematically assessed. The participants received an expense allowance of US $58. No compensations were paid in the other phases.

#### Informed Consent in Prototype Testing With Users Having Lived Experience (Phases 6 and 7)

Informed consent was obtained before participation. Participants received study information via email and confirmed their agreement in the app using checkboxes. In addition, verbal informed consent was secured before the focus groups and individual interviews.

#### Informed Consent in MVP Testing (Phase 8)

Informed consent was obtained through mandatory checkboxes during app registration, and a downloadable PDF version of the study information was provided for reference. For risk mitigation, the informed consent explained that the app did not replace psychological, psychiatric, or medical treatment, and the app contained a page with telephone numbers for current psychological emergencies.

Quantitative questionnaire data were pseudonymized during collection and anonymized before analysis.

#### Data Security and Privacy

In terms of data security, the app complied with the General Data Protection Regulation and the International Organization for Standardization 27001 standards. During prototype testing, audio recordings from Zoom (Zoom Communications, Inc) sessions were stored locally, transcribed without personal identifiers, and deleted following transcription. In MVP testing, participant data collected during registration were securely stored and removed upon permanent account deactivation or at the participant’s request. Log data were pseudonymized during data collection, and fully anonymized data were archived for research purposes after the study. Encrypted backups were stored in European Union–based data centers managed by Hetzner, with password-protected access limited to project leads. Team members signed confidentiality agreements and adhered to strict data protection protocols, ensuring no identifiable participant information was included in publications, images, or supplementary materials. Moreover, users maintained full control over their privacy and anonymity in the app. During registration, participants selected a username without any mandatory disclosure of personal information, such as age, gender, or potential diagnoses. Users decided how much they wanted to share. For example, when creating groups, they could choose whether to keep them private or accessible to others. Furthermore, the preparation and follow-up of exposure exercises could be kept private or shared with the group. This approach ensured that participants could interact in a way aligned with their comfort and well-being while safeguarding their data.

## Results

The qualitative and quantitative results of the PD study are presented subsequently in chronological order and sorted by development stages (initial app development, prototype testing, and MVP testing). The results from each stage informed the further development of the app.

### Stage 1 (Phases 1-6): Development of the App Prototype

#### Overview

On the basis of iterative co-design across phases 1 to 6, which included qualitative input from individuals with lived experience and usability feedback from students, the app was developed into three key modules: (1) psychoeducation, (2) exposure, and (3) activity scheduling. Users can access all modules simultaneously without a prescribed order of engagement, allowing for flexible navigation and personalized content selection.

#### Psychoeducation Module

The module offers concise texts and graphics as microlearning units [51], providing essential information on anxiety and therapeutic strategies such as activity scheduling and exposure. This format was designed to improve comprehension and retention [51], especially for users who reported limited time.

#### Exposure and Activity Scheduling Module

These modules are interactive and group based, directly addressing users’ requests for social support and shared accountability, enabling users to join or create groups based on shared goals. Using a private-public slider, users can name their group, add a description, upload an image, and adjust its visibility. Public groups appear in a list within the respective module, where users can preview group details and join. Upon joining, users can engage in ongoing discussions, post comments, share experiences, and provide feedback, such as fostering a collaborative and supportive community. The *activity scheduling module* helps users discover and incorporate positive activities into their daily routines. For instance, a user struggling to practice progressive muscle relaxation might join a “relaxation” group to connect with others who share practical tips, such as starting with brief sessions or using audio guides. Alternatively, users can create their own groups, such as a “yoga” group, to encourage participation in weekly sessions and track and share progress. Activity scheduling, originally derived from depression treatment [52], aims to increase engagement in pleasurable activities and promote positive environmental interactions. Research by Taylor et al [53] suggested that activity scheduling may also reduce anxiety by enhancing positive affect, cognitions, and behaviors. The peer-driven dynamic within the activity scheduling module is expected to encourage healthy behaviors, inspire users through shared experiences, and increase the likelihood of sustained engagement. Similarly, the *exposure module* supports users in confronting anxiety-inducing situations. Users can join groups with shared challenges or create their own, such as a “bus riding” group for individuals experiencing anxiety related to public transportation. Within this module, users document their experiences, for example, riding a bus, and rate their anxiety before and after completing the task. Entries can be kept private or shared with the group, allowing peer feedback and support. Group members can discuss setbacks, share successes, and offer practical advice, such as starting with short rides during off-peak hours. The exposure module is designed to systematically encourage users to confront their fears and test the validity of their anxious thoughts through behavioral experiments. According to treatment guidelines, exposure exercises are a cornerstone of cognitive behavioral therapy (for reducing anxiety symptoms and decreasing avoidance behavior in anxiety disorders [54]. Peer-to-peer interaction within this module facilitates the planning and implementation of exposure exercises, motivating users to persist through mutual accountability and emotional support. This approach empowers users, reduces avoidance behaviors, and fosters adherence to anxiety-reducing strategies.

#### Senior Role

To further enhance engagement, the app introduces a *senior role* inspired by group leaders in traditional self-help settings. Selection criteria for senior members included their personal experience with anxiety disorders, familiarity with self-help group practices, and affiliation with the German self-help organization. In addition, candidates were required to be willing to volunteer regularly. During prototype testing, participants were asked whether they could imagine stepping into a senior role, what challenges they anticipated in that position, and the support they would require. They recommended enabling direct messaging with other senior team members and emphasized the need for intervision and supervision. Senior members who supported MVP testing were test users during the prototype testing. They were familiar with the app and its functions and had contributed to refining the role of senior members. Therefore, no formal training was necessary. Once MVP testing began, senior members participated in regular supervision meetings with CZ.

The senior role was designed to minimize disruption to group dynamics while positively influencing user outcomes. Senior members were expected to intervene only in specific situations, such as providing advice upon request, assisting with task completion, or mediating conflicts. However, their role did not include offering regular feedback or one-on-one support, ensuring that the group maintained its peer-to-peer structure. By serving as accessible and equal-status points of contact, senior members modeled positive behaviors and contributed to fostering a supportive and empowering community environment.

### Stage 2 (Phases 6 and 7): Qualitative User Feedback on the Prototype

#### Overview

During this phase, the prototype of the app was tested with 9 users who were unfamiliar with the app. Through qualitative analyses of focus groups and interviews, key barriers and facilitators were identified (Textbox 1), which directly informed the refinement of app features and structure for the MVP. The first 2 main categories primarily reflected participants’ expectations and attitudes toward anticipated app use. In contrast, the remaining 4 main categories pertained to actual user experiences during the prototype testing period. Multimedia Appendix 3 provides a detailed coding system, including anchor statements.

**Outcomes from the qualitative analysis conducted during the prototype testing phase of the self-help app for anxiety. Participants (n=9) were recruited through a German self-help organization. The deductive main categories and inductive subcategories outline user feedback regarding the app’s features, design, and functionality.**

**Outcome expectation**

- Specific guidance
- Community
- Safety experience

**Interaction**

- Role of an experienced peer supporter: time management, supervision, responsibility, and psychological strain
- Interpersonal exchange: no exchange, communication appeal, and direct messaging

**Activity scheduling**

- Inspiration
- Improvement suggestions

**Exposure**

- Inspiration
- Improvement suggestions

**Psychoeducation**

- Linguistic expression
- Knowledge sharing
- Improvement suggestions: references and indexing

**User experience and functionality**

- User-friendliness
- Bugs: save and upload, edit, and navigation
- Lack of clarity
- Proposal for optimization: menu, module name, group content, optical presentation, guidance, visibility and privacy, and information flow

#### Outcome Expectations

##### Overview

Users were asked about their expectations for the app and the changes they hoped to achieve by using it. With this question, we aimed to assess users’ needs for a self-help app and how well those needs align with the app’s specific setup and content. Three main themes emerged from this.

##### Specific Guidance

Users anticipated that the app would offer structured knowledge and practical tools for managing anxiety, including step-by-step instructions for completing exercises. One participant expressed the following expectations:

[I]t was an idea to expect to...get some hints: how do I deal with anxiety in general?... Maybe skills for panic attacks are also listed, in a list or ideas or something.

B4:22

These expectations influenced updates to the psychoeducation module and exposure preparation features.

##### Community

Participants anticipated that the app would encourage mutual exchange, social support, group cohesion, and a sense of belonging among individuals experiencing anxiety. One participant shared the following:

So actually, you meet people who maybe have the same problem, and then you don’t feel so alone anymore.

B5:16

This feedback highlighted the significance of peer interactions and validated the app’s development goals in creating a supportive user community.

##### Safety Experience

Users emphasized the significance of moderated group discussions and crisis management strategies to reduce risks such as inappropriate comments or distressing situations. One participant shared the following concerns:

[B]ut in terms of content, I fear there are obscure stories in it. Sometimes, even in the group...anything like “do not put up with anything, hit back immediately!” and so on.... Then I almost fall off my chair...because I think...that’s not a positive thing.... And these are my fears, if this is not moderated, that this will come up in there...

Interview number 2:102

In response, safety features were integrated into the MVP. First, in the psychoeducation module, we included information on managing personal crises and provided a link to national crisis hotlines. Second, we established group rules, such as navigating differing opinions, offering constructive feedback, practicing self-kindness, avoiding the glorification of dysfunctional behaviors, and avoiding self-promotion and spam. Third, we introduced the role of senior members, who could be contacted for support in using the app and moderating discussions within groups. They could be reached if group rules were violated or when conflicts arose. Fourth, we developed a reporting tool that enabled every user to flag inappropriate content. Flagged content became invisible until a senior peer decided to address or remove it. Senior members could consult one another before acting. This system was designed to ensure user safety and distribute responsibility among group members, thereby reducing the burden on individual senior members (refer to the subsections Responsibility and Psychological Strain).

#### Interaction

We asked about the app’s specific functions and modules to identify what additions or changes might be beneficial as well as which features were viewed as especially useful. In addition, we explored the role of senior members and any challenges related to their position.

##### Role of Experienced Peer Supporters

Participants were asked about their perceptions of a senior peer as a guide within the app as well as any challenges they might face.

##### Time Management

Users emphasized the necessity for clearly defined response times to manage expectations about senior availability:

[S]o I would say that there should be the possibility that you can...write in [the app] at any time of day or night, but the seniors’ reaction...that maybe needs to be set to a time frame.

Interview number 3:50

##### Supervision and Guidance

Users highlighted the importance of providing senior members with continuous feedback and support to manage complex situations.

##### Responsibility

Participants raised concerns regarding senior members’ responsibility for app-generated content. In response, the MVP clarified that senior members were not required to monitor discussions continuously and were supported through intervision and supervision (Stage 1 [Phases 1-6]: Development of the App Prototype section). As the user base grows, additional senior members will be recruited. Prospective candidates must meet specific eligibility criteria, including a minimum registration period, and complete a structured training program before assuming the senior role.

##### Psychological Strain

Participants expressed concerns about the potential stress linked to being a senior member, fearing it could lead to pressure and anxiety:

Do I feel responsible for what comes up in the chat room or in the exchange at all?... Do I FEEL responsible there? And this can...create pressure, stress, and anxiety...

Focus group 1:162

To address this matter, explicit role definitions and supportive mechanisms, namely intervision and supervision, were established.

##### Interpersonal Exchange

Although the prototype did not fully integrate various communication modes, participants shared their thoughts on in-app communication, emphasizing its benefits and challenges. Users expressed a substantial interest in direct communication options, highlighting the need for effective messaging features. These findings reinforced the ongoing efforts to develop a consistent messaging system.

#### Feedback on App Modules

##### Overview

Participants assessed the modules centered on activity scheduling, exposure, and psychoeducation. We aimed to understand how participants, who were experts with lived experience but were not part of the initial app development, perceived the importance and practical application of the modules. Key findings are given in the subsequent sections.

##### Activity Scheduling and Exposure

Users appreciated the ability to draw inspiration from their peers’ exercises and valued the feature that allowed them to rate their anxiety levels both before and after exposure. This functionality helped contextualize their personal experiences and normalize anxiety:

Yes...I think it makes sense. I think that simply recording fear triggers [helps],...it is often the case that I have a fear and think to myself, “I am weird, no one else has that”...

Interview number 4:48

On the basis of the feedback in this category, the preparation and follow-up sections of exposure exercises were refined to improve clarity and understanding.

##### Psychoeducation

Although users generally appreciated the psychoeducational content, they suggested simplifying the texts for better understanding.

#### Usability, Navigation, and Function

From the feedback gathered, 3 main themes emerged.

##### Technical Issues

The most frequently reported issue was fixing bugs, especially those related to saving posts, uploading photos, and editing user comments.

##### Navigation

Users expressed a desire for improved navigation within the app. They often found themselves seeking clearer guidance on the actions to take and the appropriate moments to undertake those actions. They suggested integrating a well-structured user journey in the MVP to address this confusion and promote greater compliance. A recurring theme in their feedback was the arrangement of the app’s modules. Users believed that a more logical flow would substantially enhance their experience. For instance, they recommended moving the exposure module to precede the activity scheduling module, enabling users to transition smoothly between tasks. In addition to reorganizing the modules, users expressed a preference for renaming them to make them more relatable. They proposed changing “activity scheduling” to something such as “what helps me” and suggested replacing “psychoeducation” with a simpler term such as “wiki.” They felt these changes would resonate better with users and make navigation more intuitive. Furthermore, they suggested adding a sorting function to simplify the activity scheduling process. This feature would help users easily identify groups that match their personal interests and preferences, ultimately fostering a more personalized and engaging experience with the app.

##### Information

Participants emphasized the importance of information buttons, pop-ups, reminder functions, and a progress-tracking page to display exercises, along with detailed descriptions of the purposes and benefits of modules and groups. These features were deemed essential for enhancing task comprehension and potentially increasing user engagement and participation. A commonly suggested improvement was the ability to preview group descriptions before joining, enabling users to make more informed decisions. Participants also highlighted the need for better visualization of exposure exercises, such as graphical representations that showed how their experienced anxiety was lower than expected. In addition, they requested a broader variety of profile pictures for groups, possibly by incorporating stock images.

#### Additional Steps

Qualitative feedback informed further development of the prototype before it was tested in 2 focus groups with 9 students to explicitly test and enhance user-friendliness.

##### Key Areas for Improvement

Students and test users highlighted the necessity for clearer explanations of the app’s core content and features to improve usability. Their recommendations centered on refining the navigation bar layout, providing concise instructions within modules to clarify their purposes, and adding notifications to keep users updated about new messages and events. However, opinions differed regarding the effectiveness of notifications as motivational tools, with some participants expressing concerns that these might create unnecessary pressure.

##### Usability and Technical Improvement

Students suggested creating preexisting example groups to illustrate proper use of exposure and activity scheduling modules, implementing a messenger-style interface that allows direct replies to specific messages for improved communication, streamlining the psychoeducation module with graphics and expandable sections to support the microlearning approach, enabling progress tracking for completed tutorials and beneficial activities, and developing user profiles to foster more interactions about personal experience.

##### Adjustment of App Development Process

Considering the extensive feedback and challenges faced during prototype testing, we decided to modify the development process by narrowing the scope of future iterations to prevent unnecessary programming, collecting feedback at shorter intervals to ensure it meets user needs, and adding a reporting feature for users to directly report technical issues.

##### Significance of Student Testing

The dual feedback approach provided essential insights from various perspectives. Test users with lived experience evaluated whether the app’s content met their expectations and identified potential barriers to adapting the self-help model to an online format. Students primarily assessed usability and technical execution, confirming that previously reported issues were resolved and identifying new technical and comprehension challenges. By incorporating feedback from both groups, we ensured a broader range of perspectives, including those from both technologically proficient users and less experienced individuals. In addition, students served as a readily accessible and impartial test group compared to members of the German self-help organization.

### Stage 3 (Phase 8): Quantitative Results of MVP Testing

#### Overview

Following the qualitative feedback obtained from individuals with lived experience and students (as described in the earlier phases), the app prototype was revised to address specific usability concerns. Improvements were made to simplify navigation, clarify module instructions, particularly for exposure exercises, and include supportive group functions. These changes were integrated into the MVP, which was made publicly accessible via the German self-help organization’s website for broader testing.

#### Demographic Characteristics

Baseline data were collected from a sample of 20 users who completed pretreatment questionnaires. Table 1 presents the demographic characteristics of the sample.

**Table 1:** Overview of the demographic characteristics of test users who provided baseline data for testing the minimum viable product (n=20).

Variable		Users, n (%)
**Age (y)**		
	18-24	0 (0)
	25-34	6 (30)
	35-50	10 (50)
	>50	4 (20)
**Gender**		
	Women	15 (75)
	Men	5 (25)
**Marital status**		
	Married	10 (50)
	Divorced	3 (15)
	Single	7 (35)
**Self-reported mental disorder diagnoses**		
	Generalized anxiety disorder	9 (45)
	Panic disorder	2 (10)
	Remaining diagnoses (depressive, alcoholic, or other disorder)	9 (45)
**Treatment history**		
	Outpatient psychotherapy	11 (55)
	Inpatient or partial inpatient therapy	2 (10)
	Self-help groups	1 (5)
	No previous treatment	5 (25)
	Other treatment form	1 (5)

#### MVP Use and Engagement Patterns; Participant Flow and Dropout

**Figure 2:**
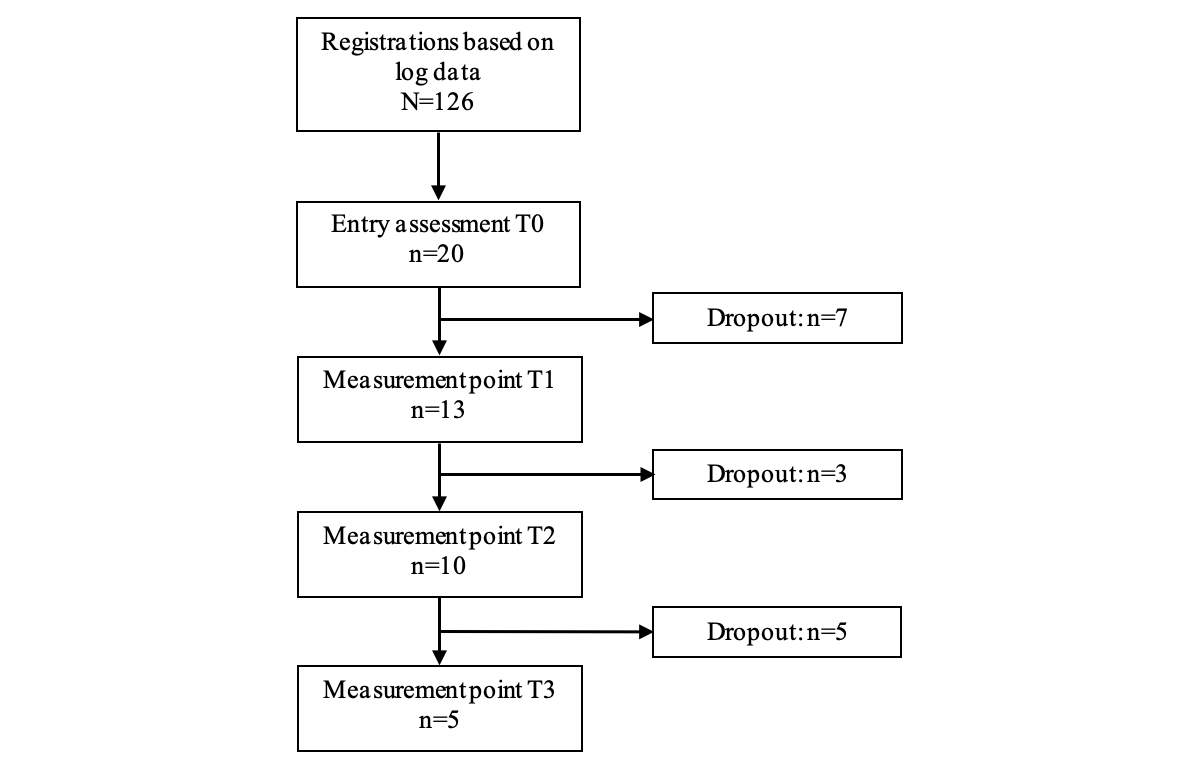
Flowchart illustrating the quantitative data collection process during the minimum viable product (MVP) testing phase for the self-help app aimed at alleviating anxiety. A total of 126 participants were involved. Measurement points included baseline (T0) and 4 (T1), 8 (T2), and 12 weeks (T3), with questionnaires evaluating app use, adherence, and user experience. Dropout rates steadily increased across the measurement points.

Despite targeted design improvements, log data indicated persistent engagement issues. Of the 126 registered users, only 20 (15.9%) completed the baseline questionnaires, and substantial dropout occurred over time, with 75% (15/20) of the participants failing to respond at T3 (Figure 2).

This limited engagement reflected the qualitative findings where users reported confusion, emotional overload, and a lack of structured support, particularly in the exposure module. For example, several interviewees shared uncertainty about how to perform exposure exercises. This aligned with the observation that only 25% (5/20) of the survey participants engaged with the exposure module, and only 2 participants from the entire sample sent any messages via the platform.

#### Outcome Questionnaires and Relation to App Use

The mean anxiety scores at T0 (GAD-7: mean 10.52, SD 5.15) reflected moderate impairment, consistent with the target population. According to established clinical cutoffs [43], 9% (2/21) of the participants were classified as experiencing minimal strain, 43% (9/21) as experiencing mild strain, 24% (5/21) as experiencing moderate strain, and 24% (5/21) as experiencing severe strain. The MHSE scores seemed comparable to normative data in clinical samples [49] and improved over time (from a mean of 32.85, SD 12.94 to a mean of 43.83, SD 12.50), suggesting a possible link between active use, especially of the activity scheduling module, and perceived coping ability. This module also had the longest average use time of 8.34 minutes, indicating that users found it both accessible and helpful. Qualitative feedback corroborated this, as users highlighted that “concrete activity suggestions felt more motivating than educational texts.” The brief measure of general self-efficacy (ASKU) showed that participants felt moderately self-efficacious (mean 3.10, SD 0.90), with a nonclinical sample reporting slightly higher average scores. Conversely, general well-being (WHO-5) slightly decreased at T3 (from a mean of 15.80, SD 6.17 to a mean of 12.70, SD 5.85), which may reflect reduced overall engagement or increasing dropout over time. Using nonclinical cutoffs [46], 10% (2/20) of the participants were classified as having substantially reduced well-being, 20% (4/20) had reduced well-being, 25% (5/20) reported satisfactory well-being, and 45% (9/20) reported very good well-being. Participants reported low perceived social support (Oslo Social Support Scale-3: mean 7.25 SD 2.67), aligning with both quantitative results and qualitative concerns about the usefulness of peer group features. Although many participants joined groups for activity scheduling (9/20, 45% among survey participants), almost none initiated or actively communicated within these groups, highlighting a persistent challenge in fostering interactive support environments. Among participants, 0% (0/10) were classified as having strong social support, 30% (3/10) had moderate social support, and 70% (7/10) had poor social support. Life satisfaction, as measured by the L-1, was low on average (mean 4.15, SD 2.08; Table 2).

**Table 2:** Summary of the quantitative results for the minimum viable product. It displays survey data collected at various time points (baseline time point [T0], 4-week time point [T1], 8-week time point [T2], and 12-week time point [T3]) for different mental health and well-being indicators. Classification scales are used to categorize the results for each indicator.

Scale and comparative scores	T0, mean (SD)	T1 (SD)	T2, mean (SD)	T3, mean (SD)	Scores, mean (SD)
GAD-7^a^ (0-4: minimal anxiety, 5-9: mild anxiety, 10-14: moderate anxiety, 15-21: severe anxiety)	10.52 (5.15)^b^	13.30 (4.35)^c^	11.45 (5.99)^d^	—^e^	2.95 (3.41)^f^
WHO-5^g^ (19-25: very good, 13-18: overall satisfactory, 12-10: reduced, 10>: significantly reduced)	15.80 (6.17)^h^	18.70 (3.74)^c^	17.18 (5.17)^d^	12.70 (5.85)^i^	17.58 (4.97)^j^
MHSE^k^	32.85 (12.94)^h^	35.11 (8.57)^l^	37.27 (14.56)^d^	43.83 (12.50)^i^	33.23 (11.45)^m^
ASKU^n^	3.10 (0.90)^h^	2.96 (0.48)^l^	3.50 (0.96)^d^	3.83 (0.86)^i^	4.00 (0.74)^o^
OSSS-3^p^ (3-8: poor support, 9-11: moderate support, 12-14: strong support)	7.25 (2.67)^h^	7.78 (2.17)^l^	9.00 (2.49)^d^	8.83 (2.40)^i^	10.16 (2.07)^q^
L-1^r^ (0: not at all satisfied, 10: completely satisfied)	4.15 (2.08)^c^	3.70 (1.25)^c^	4.27 (3.07)^d^	5.83 (1.47)^i^	7.18 (2.07)^s^

^a^GAD-7: Generalized Anxiety Disorder-7.

^b^n=21.

^c^n=10.

^d^n=11.

^e^Not available; no calculations could be performed due to missing data (dropout).

^f^Löwe et al [44] and Spitzer et al [43].

^g^WHO-5: World Health Organization-Five Well-Being Index.

^h^n=20.

^i^n=6.

^j^Brähler et al [46].

^k^MHSE: Mental Health Self-Efficacy Scale.

^l^n=9.

^m^Clarke et al [49].

^n^ASKU: Short Scale for General Self-efficacy Beliefs.

^o^Beierlein et al [47].

^p^OSSS-3: Oslo Social Support Scale-3.

^q^Kocalevent et al [50].

^r^L-1=General Life Satisfaction Short Scale-1.

^s^Beierlein et al [48].

#### System Use: Full Sample Versus Survey Participants

A total of 125 participants (excluding senior members and 1 individual whose log data were not saved) logged into the app, with an average of 3.15 (SD 14.37) visits per participant. Among these, 86 participants (68.8%) accessed the app only once after registration, 18 (14.4%) visited twice, 13 (10.4%) visited 3 times, and 8 (6.4%) visited ≥5 times.

Regarding messaging activity, 123 (98.4%) of the 125 participants sent no messages, while 2 (1.6%) sent ≥2 messages. The average page hits per visit were 32.39 (SD 28.49*).* On average, participants engaged in activities on 2.02 (SD 4.94) days. The average active time spent was 6.02 (SD 10.57) minutes in the psychoeducation module, 1.55 (SD 2.43) minutes in the exposure module, and 8.34 (SD 32.28) minutes in the activity scheduling module.

Regarding group participation, 79 (62.7%) of the 125 individuals joined ≥1 exposure group, while 2 (1.6%) created a new exposure group. In total, 104 (82.5%) participants joined ≥1 group for activity scheduling, and no (0%) participant created any activity-scheduling groups.

The average number of visits among the 20 survey participants was 2.60 (SD 2.19). Specifically, 7 (35%) participants visited the app only once after registration, 7 (35%) visited twice, 3 (15%) visited 3 times, and 3 (15%) visited ≥6 times. None of the participants sent messages. The average number of page hits per visit was 31.95 (SD 21.37). On average, participants engaged in activities on 2.30 (SD 1.81) days. The average active time spent in the psychoeducation module for 16 participants was 7.42 (SD 10.51) minutes; in the exposure module for 15 participants it was 1.34 (SD 1.29) minutes; in the activity scheduling module for all 20 participants it was 9.27 (SD 12.21) minutes*.* Regarding group participation, 5 (25%) participants joined ≥1 exposure group, and none created a new exposure group. For activity scheduling, 9 (45%) participants joined ≥1 group, and no participants created any activity scheduling groups.

#### Integrated Interpretation and Implications for Further Development

The MVP of the self-help app was refined based on qualitative feedback from individuals with anxiety and students, addressing issues such as unclear structure, a lack of motivation, and confusing modules. These changes aimed at enhancing usability and engagement. Despite these improvements, quantitative data indicated that user engagement remained low: only 15.9% (20/126) of the registered users completed the baseline survey, and 75% (15/20) dropped out by T3. Most (7/20, 35%) users logged in only once, and very few interacted with group features and none sent messages, mirroring earlier qualitative reports of confusion and insufficient guidance, particularly regarding the exposure module. Outcome measures revealed moderate anxiety and self-efficacy at T0. While self-efficacy increased over time, especially in modules such as activity scheduling, overall well-being and life satisfaction remained low, likely due to limited engagement. Qualitative and quantitative findings consistently highlighted the need for better onboarding, clearer structure, and more support to improve adherence and therapeutic effectiveness.

## Discussion

### Principal Findings

This study aimed to investigate the development of a self-help app using the IDEAS framework as a guiding structure rather than as a strictly linear road map. Overall, the findings indicate that, despite the extensive involvement of experts with lived experience, a stepwise and framework-informed development approach, and repeated mixed methods data assessments, the app was not acceptable to individuals seeking self-help for anxiety. Efficacy could not be estimated due to low use. Nevertheless, this study provides valuable insights into user engagement, adherence, and the challenges of creating a peer-supported digital intervention for anxiety.

### Participatory Intervention Development

The app’s development actively engaged software engineers, members of a self-help organization, and psychologists. This multidisciplinary collaboration aimed to ensure that the intervention was both technically feasible and responsive to the needs of individuals with anxiety. Although stakeholders’ involvement enriched the content and functionality, several challenges emerged, particularly in aligning diverging priorities within a limited time frame, which often affects the thoroughness of analysis and preparation between user feedback sessions [37]. Such difficulties are common, as varied professional backgrounds can lead to task conflicts. Remarkably, these conflicts often yield more innovative and effective outcomes [55] when managed effectively.

In this study, the limitations of time led to the initial prototype featuring only a small set of functions and several unresolved usability issues. External test users in prototype testing (phases 6 and 7) reported fundamental difficulties in understanding and navigating the app. These late-stage insights were highly relevant but arrived too late to inform earlier design decisions meaningfully. Consequently, the MVP of the app included only a limited number of features and retained unresolved barriers to user engagement. This led to minimal messaging and low overall activity within the app, findings that directly relate back to these development gaps. Future iterations should integrate usability testing, including participants with minimal previous experience in digital self-help, to ensure that app structures are intuitive and actionable for the intended audience.

### Adherence and User Engagement

As demonstrated by baseline data, the MVP successfully attracted its intended target group, individuals experiencing anxiety and low social support. However, true engagement with the intervention remained low. Log data revealed that many users accessed the app only once, logged in infrequently, rarely communicated, and did not actively participate in scheduling activities or exposure-related group tasks. This lack of adherence raises substantial concerns about the feasibility of a fully self-guided peer-support approach without professional facilitation.

A particularly striking result was the near-complete absence of messaging activity; although 63.2% (79/125) of the participants joined an exposure group and 83.2% (104/125) joined an activity scheduling group, 98.4% (123/125) did not send a single message. This suggests that participants were curious enough to explore group features but lacked the confidence, clarity, or motivation to engage in communication, an essential mechanism of peer support. Qualitative feedback further highlighted barriers such as uncertainty about message visibility, fear of doing something wrong, and the absence of initial engagement from others. One interviewee explained the following:

[T]he problem was probably that there were simply no comments or feedback, so I had to make the first step, and then I realized...this is visible to all. What if I do something wrong?

Interview number 1:27

These findings suggest that joining a group does not equate to meaningful participation. While similar issues have been reported in previous studies [56-58], the level of engagement observed in this study was comparably lower. For instance, Hanano et al [57] conducted a secondary analysis of an RCT on a self-guided intervention for anxiety and depression and found an average of 2.14 logins per module across the 8 modules available, with 48% to 63% of the participants not logging in at all. Similarly, another RCT evaluating an online intervention for treating depression, which included interactive features, such as animation, video, and narration, recorded an average of 18.7 logins per participant [56]. By contrast, in this study, the mean number of logins was only 3.15 (SD 14.37), with many (125/125, 100%) users logging in just once.

In terms of page views, our findings (mean 32.39, SD 28.49) were comparable to those of another self-guided intervention aimed at reducing social anxiety symptoms, which reported an average of 37.6 (SD 41.3) page views [58]. However, this apparent similarity requires critical interpretation, as the intervention followed a structured, sequential module design, offering clearer user pathways and guidance. In contrast, our app featured an open structure without step-by-step progression or automated guidance, potentially limiting user orientation and reducing sustained engagement. The comparison underscores a critical insight; that is, while peer support is theoretically promising, it may not emerge spontaneously in an unmoderated digital environment. This app was designed with an open structure that does not dictate a specific sequence of interactions or instructions on use. Without consistent user engagement, the core principle of peer support cannot be realized, leading to reduced interaction, less communication, and a weakened sense of community. In turn, this leads to user attrition as group cohesion fails to develop.

Technical challenges and usability issues likely compounded these difficulties and contributed to dropout [27]. Despite efforts to improve navigation and clarify app functions during development, user feedback indicated ongoing confusion and anxiety about how the app was supposed to work. This is consistent with the systematic review by Borghouts et al [59] of user engagement with digital mental health interventions, which found that technical difficulties and privacy concerns are key predictors of disengagement. This aligns with feedback from our test users, who reported difficulties with the app’s complexity and a lack of transparency regarding its functions and tasks, which hindered their engagement. To address these issues, efforts were made to improve the app’s intuitiveness by introducing a clearer structure and a user journey to enhance navigation.

Overall, our findings indicate that an open, nonsequential peer support model may create entry barriers too high for meaningful user participation. Consequently, the app did not create an environment conducive to users learning from one another [60,61], experiencing empowerment, and enhancing their self-efficacy in anxiety management [4]. Unlike previous research [62,63], our results did not confirm that peer support can be a substitute for professional guidance or substantially enhance adherence. Therefore, future versions should include structured onboarding and behavioral guidance, such as short interactive tutorials, step-by-step introductions to key features, or even temporary facilitation (eg, peer moderators or bots), to encourage interaction and support early-stage engagement while building initial trust, particularly for users with low digital confidence.

### Anxiety and Self-Efficacy

Due to low adherence, inferential statistics were not used. However, descriptive data revealed only minimal changes in anxiety levels (GAD-7), suggesting that the intervention had limited impact on symptom reduction. Similarly, general self-efficacy scores showed negligible changes in mean values. However, mental health–specific self-efficacy showed a somewhat more pronounced improvement (MHSE: mean 42.83, SD 12.50) compared to clinical reference values (mean 33.23, SD 11.45) [49]), indicating that even minimal peer-based digital interactions might offer motivational effects. While this increase is promising, it should be interpreted with caution due to limited use and the lack of inferential testing; these factors highlight the limited effects in the context of low user engagement. Because the app relied heavily on peer interaction and user engagement, insufficient communication combined with minimal repeated use likely hindered the intended changes from occurring. Specifically, features such as exposure group participation and activity scheduling were seldom used effectively, often lacking peer dialogue or follow-up actions. In the absence of active involvement or feedback from peers, chances for collaborative learning, emotional support, and reinforcement of coping strategies were mostly missing. The slight increase in mental health–specific self-efficacy may reflect a general sense of motivation or positive expectation following onboarding. However, this optimism did not translate into sustained interaction with the app, as shown by use patterns. One possible interpretation is that some users initially believed the app could help but became disengaged when faced with unclear instructions, a lack of guidance, or low peer activity, dampening the motivational effect over time.

Future research should focus on strategies to enhance the app’s attractiveness and usability to bridge the effectiveness gap. Potential upgrades may involve setting up exposure exercises in advance to clarify their application, encouraging active engagement among senior members, or creating a buddy system to boost user interaction.

### Sample Characteristics

The sample predominantly consisted of individuals aged >35 years (14/20, 70%), many (19/20, 95%) of whom reported no previous experience with self-help. Although not statistically tested in this study, age and previous experience may have influenced digital engagement. Older users, particularly those unfamiliar with digital platforms, may have required more structured onboarding and clearer guidance to navigate the app effectively. This was also reflected in usability feedback during prototype testing, where several participants expressed confusion regarding the app’s functions, even though they were experienced with self-help. These factors likely contributed to the app’s limited use, as shown by log data.

### Limitations and Future Directions

This study has several limitations that should be acknowledged when interpreting the results and considering their generalizability. First, all participants for prototype testing were recruited from a specific German self-help association and had previous experience with face-to-face self-help. In contrast, MVP participants were recruited via the association’s website without specific inclusion criteria. This recruitment strategy likely introduced a selection bias favoring individuals with a positive predisposition toward self-help and a higher digital affinity. Therefore, the findings may not represent individuals who are less motivated, less digitally literate, or skeptical of peer-based approaches. Future studies should aim to diversify recruitment by engaging participants from clinical settings, community organizations, and social media platforms to include a broader spectrum of digital experiences and attitudes toward self-help. Second, the regional focus on German-speaking users limits the generalizability of the findings across various cultural and linguistic contexts. Attitudes toward mental health, help-seeking, digital interventions, and peer support may vary substantially among countries, potentially influencing engagement and effectiveness. Therefore, future research might include culturally adapted versions of the app and recruit participants from different linguistic and cultural backgrounds. Third, while 126 individuals registered for MVP testing, only 86 (68.3%) logged in at least once. Moreover, a substantial proportion did not complete the baseline assessments, and approximately 75% (15/20) of the active users dropped out by T3. This high attrition rate resulted in substantial missing data and reduced the ability to assess long-term usability, engagement, or symptom changes. In addition, the limited follow-up undermined statistical power and internal validity. Researchers should implement targeted retention strategies (eg, regular reminders) to reduce attrition in future studies and ensure good usability. Furthermore, qualitative check-ins or exit interviews could also help capture the reasons for dropout. Fourth, although 63.2% (79/125) of the users joined peer groups for exposure exercises and 83% (104/1125) joined peer groups for activity scheduling, few actively engaged with the group features. This raises concerns about the distinction between initial interest and sustained behavioral engagement. Future studies should explore mechanisms to enhance group involvement, such as onboarding tutorials, group moderators, gamification elements, and tailored content recommendations. Collecting early qualitative feedback on perceived barriers to group participation would help improve peer interaction features and inform design changes that foster stronger community ties. Ultimately, the absence of a control group or randomization prevents causal conclusions regarding the app’s effects. Without comparative data, it remains uncertain whether the observed patterns stem from the intervention itself or from external factors. To address this, future research should use RCT designs and consider using mixed methods approaches to better grasp both quantitative and qualitative outcomes.

### Conclusions and Implications

This study contributes to the field of digital mental health research by evaluating the strengths and weaknesses of participatory methods in creating a peer-guided self-help app for managing anxiety. Involving users with personal experience in anxiety was beneficial, but the results challenge the assumptions of social identity theory. They reveal that shared digital environments do not automatically foster a sense of community or user engagement without adequate support and moderation. Furthermore, the research emphasizes the importance of consistently comparing user feedback with actual use data and technical capabilities during the app’s development. Issues such as high dropout rates, limited understanding of why users leave, and low interaction with features highlight fundamental design and implementation struggles digital self-help tools face. These findings stress the need for well-organized onboarding processes, clear interaction prompts, and transparent moderation policies to minimize user uncertainty. By pinpointing key challenges in process and design, this study offers valuable insights for creating user-centered digital interventions not just for anxiety self-help but also for other peer-supported or community-driven mental health initiatives. Looking ahead, future studies should focus on refining technology, broadening recruitment efforts, and conducting early user testing to improve overall effectiveness.

## Appendix

Multimedia Appendix 1 Semistructured focus group interviews.

Multimedia Appendix 2 Semistructured individual interviews.

Multimedia Appendix 3 Coding system.

## Abbreviations

ASKU: Short Scale for General Self-efficacy Beliefs
GAD-7: Generalized Anxiety Disorder-7
IBI: internet-based intervention
IDEAS: integrate, design, assess, and share
L-1: General Life Satisfaction Short Scale-1
MHSE: Mental Health Self-Efficacy Scale
MVP: minimum viable product
PD: participatory intervention development
RCT: randomized controlled trial
T0: baseline time point
T1: 4-week time point
T2: 8-week time point
T3: 12-week time point
WHO-5: World Health Organization-Five Well-Being Index

## Data Availability

The datasets generated or analyzed during this study are available in the Open Science Framework repository [64].
