# Supplementary material for: Participatory Intervention Development of a Peer-Guided Self-Help App for Anxiety Disorders: Mixed Methods Study
Source: JMIR Form Res. 2025 Jun 20;9:e62781. doi: 10.2196/62781 (PMC12228002; doi:10.2196/62781)
Supplement: Multimedia Appendix 1 [file formative_v9i1e62781_app1.docx]

| **1. Welcome and introductory words** |
| --- |
| **2. Questions** |
| Outcome expectations |
| - What did you expect from the app when you started using it? - What should change for you as a result of using the app? |
| Impact & benefits (general strengths and weaknesses) |
| - What do you think are the biggest strengths & weaknesses of the app? - Do you have any suggestions on how to fix the weaknesses? - How big is the difference between what you expected and what the app gave you? |
| Exchange & communication (general) |
| - How did you experience the exchange with others in the app? - What was good? - What was difficult or kept you from using it?   There are plans to add a 'senior' feature to the app. Seniors would be people who help users with tips. You all would be candidates for this since you already have experience with support groups.   - Would the senior function be helpful? - What do you expect in terms of burden for yourselves or other 'seniors’? |
| Function: Behavioral activation |
| - What do you think of behavioral activation? - What did you enjoy? - What was difficult or disruptive? What kept you from using this feature? - Is there anything missing? |
| Function: Exposition |
| - What do you think about exposition exercises? - What did you enjoy? - What was difficult or annoying? What kept you from using this function? - Is there anything missing? |
| Function: informational texts |
| - What do you think of the information texts? - What don't you like about the function? What kept you from using it? |
| Layout, navigation & functionality |
| - How intuitively can you navigate through the app? - Were there any difficulties in using the app? - What made it difficult for you to use the app?   Usability with validated instrument 🡪 SUS |
| So many suggestions. We will incorporate everything.  We will send out summaries. Feel free to send comments and improvement ideas to us |
| Farewell and Outlook |
| - THANK YOU! - Inquiries gladly per email |
