# Supplementary material for: Participatory Intervention Development of a Peer-Guided Self-Help App for Anxiety Disorders: Mixed Methods Study
Source: JMIR Form Res. 2025 Jun 20;9:e62781. doi: 10.2196/62781 (PMC12228002; doi:10.2196/62781)
Supplement: Multimedia Appendix 2 [file formative_v9i1e62781_app2.docx]

| **1. Welcome and introductory words** |
| --- |
| **2. Questions** |
| Outcome expectations |
| - What did you expect from the app? - What should change for you as a result of using the app? |
| Impact & benefits (general strengths and weaknesses) |
| - What do you think are the biggest strengths & weaknesses of the app? - Do you have any suggestions on how to fix the weaknesses? - How big is the difference between what you expected and what the app gave you? |
| Exchange & Communication |
| - How did you experience the exchange with the others in the app? - What did you find pleasant or good? - What was difficult or prevented you from using the app?   There are plans to integrate a 'senior' function into the app. Seniors would be people who assist users* with tips. You would also be a candidate for this since you already have experience with support groups.   - Would a senior function be helpful? - What do you expect to be the burden should you take on a senior role? |
| Function: Behavioral activation |
| - What do you think of behavioral activation? - What is pleasant or good? - What is difficult or disruptive? What keeps you from using this feature? - Is there anything missing?   Now a few more questions about your personal experience....   - Did you prefer to do the behavioral activation alone or with others? - How did it feel to commit to this exercise in the app? - Was there anything you found particularly helpful? |
| Function: Exposition |
| - What do you think of exposition exercises? - What is pleasant or good? - What is difficult or annoying? What kept you from using this feature? - Is there anything missing?   Now a few questions about your personal experience...   - How was it to do the exposition exercise without direct support? - How did it feel to commit to these exercises in the app? - Was there anything you found particularly helpful? |
| Function: informational texts |
| - What do you think about the information texts? - What did you (not) like about the function? What kept you from using the function? |
| Layout, navigation & functionality |
| - How intuitively were you able to navigate through the app? - Were there any operating difficulties at any points? - What made it difficult for you to use the app?   Usability with validated instrument 🡪 SUS |
| Farewell and outlook |
| - THANK YOU! - Inquiries gladly per email |
