# Supplementary material for: Participatory Intervention Development of a Peer-Guided Self-Help App for Anxiety Disorders: Mixed Methods Study
Source: JMIR Form Res. 2025 Jun 20;9:e62781. doi: 10.2196/62781 (PMC12228002; doi:10.2196/62781)
Supplement: Multimedia Appendix 3 [file formative_v9i1e62781_app3.docx]

| Level 1 | | Level 2 | Level 3 | Description | Example of anchor |  |
| --- | --- | --- | --- | --- | --- | --- |
| 1. Expectation | | 2.1. Specific guidance |  | - Receive concrete guidance during exercises - Acquiring expertise | "(...) for example, [if] you feel anxious and panicky, who then goes like this for five minutes: "Now breathe in, breathe out". |  |
| 2.2. Community |  | - Expectations of mutual exchange of information - Social support - Community experience - Motivation increase - Access to information websites, linking, networking | "(...) if there (...) are enough people online, then (...), you might always find someone who then writes: "Yes, you can do it" or something." |  |  |  |
| 2.3. Safety experience |  | - Statements regarding safety during app use - Control instances within the app - Emergency assistance - Moderation | "(...) Well, that would reassure me if I knew that (...). That I as a participant don't have to worry, for example, if someone writes: "I want to kill myself". (...) That there [is] just such a moderation, (...) against the evil internet insults and (...) if [one] feels bad and so." |  |  |  |
| 2. Interaction | | 2.1. Role of experienced peer supporter |  | - Statements on the senior function - Perception of senior tasks | ---------- |  |
| 2.1.1. time management | - Time management in the performance of duties as a senior | "(...) I'm on vacation now and say, "I'm not up for psycho stuff now," (...) and I'm away for two weeks, then the users don't get anything out of it when I respond to a topic that's already two weeks old, for example." |  |  |  |  |
| 2.1.2. Supervision | - Desire for supervision - Rules of conduct in fulfilling the senior role | "(...) if you (...) are a moderator, that you have (...) a feedback (...). Or (...) supervision (...), if something triggers you (...)". |  |  |  |  |
| 2.1.3. Responsibility | - Position of the senior in relation to the users - Verantwortung für Inhalte der einzelnen Gruppen | "(...) Do I feel responsible for what comes up in the chat room or the exchange? (...) And this can of course also create pressure, stress, and anxiety." |  |  |  |  |
| 2.1.4 Psychological strain | - Fears of psychological stress due to the senior role | "(...) I would have little fear that (...) someone would pull me down. I'm used to that from the support group, and it doesn't happen that way, at least with me." |  |  |  |  |
| 2.2. Interpersonal exchange |  | - Erwartungen an den interpersonellen Austausch in der App | ---------- |  |  |  |
|  | 2.2.1. No exchange | - Lack of communication in the app | "(...) I think (...) that we were too few (...). Then I somehow thought: Well, he has now created a group, why doesn't he write anything? (...) I saw that something had been created, but (…) why should I be the first to write something? (...)" |  |  |  |
| 2.2.2. Communication appeal | - Suggestions for improving contact within the app | "(...) So I don't know, I was missing something like (...) on WhatsApp "So and so has created a group" and then there's usually a "Hello" at least (...)" |  |  |  |  |
| 2.2.3 Direct messages | - Enabling direct exchange between group members | "(...) there would still be the possibility that a chat function is installed in some way, then you could also request the chat." |  |  |  |  |
| 3. Activity scheduling | | | 3.1. Inspiration |  | - Aspects of the behavioral activation exercises users liked | "(...) you can look at what is good for others (...), [that] is just brilliant, because I don't think you come up with many things yourself that can help you with anxiety. (...)" |
|  |  |  | 3.2. Improvement suggestions |  | - Suggestions for improvement of behavioral activation exercises | "(...) whether [the groups] won't become too many at some point? Whether you could somehow combine them into groups? There are not too many yet, but if everyone puts something in?" |
| 4. Exposure | | 4.1 Inspiration |  | - Aspects of exposition exercises that user liked | “I think the idea is great. Because (...) you could see (...): What kind of development is taking place there, right?" |  |
|  |  | 4.2. Design of exposure |  | - Negative aspects of the use of courage exercises | "(...) I just think this text, "What do you avoid if possible?", that's ONE form of fear and (...) how I deal with it. But there are also situations in which I can no longer avoid. So, there I am suddenly in the fear, whether I want or not, and cannot get away from it." |  |
| 5. Psychoeducation | | 5.1. Linguistic expression |  | - Negative aspects of tutorials | "(...) But I think especially new people who have never read anything about [anxiety], when they read "with self-blame", that can be misunderstood. So, I don't know if you want to write it that way." |  |
|  |  | 5.2 Knowledge sharing |  | - Aspects that users like and that worked well | "(...) the tutorials are definitely REALLY cool, (...) the different directions of fear are described, I find very, very good. (...)" |  |
|  |  | 5.3 Improvement suggestions |  | - Recommendations of the users, for the improvement of the information texts | ---------- |  |
|  |  |  | 5.3.1. References | - Request for source citation in the tutorials | "So, I would still be interested in who researched and wrote these texts in the first place?" |  |
|  |  |  | 5.3.2. Indexing | - Linking tutorials with behavioral activation and exposition exercises | "(...) in this exposition group, I could get a selection of keywords, or I could have access to keywords that are then eventually linked or linked to the appropriate tutorials (...)." |  |
| 6. User experience & Functionality | 6.1 User-friendliness |  | - Positive feedback on the ease of use of the app | (...) the ease of use is good, for this version. So, I mean, it's not finished yet, (...)." |  |  |
| 6.2 Bugs |  | - Programming errors that prevent users from using the program as intended. | ---------- |  |  |  |
|  | 6.2.1 Save or upload | - Problems uploading or saving photos and posts | "(...) In my own opinion, I would also have added my two cents to a contribution, and somehow I can't find it anymore (...)." |  |  |  |
|  | 6.2.2 Edit | - Editing of foreign contributions is possible | "(...) I could have deleted the comment from that person. And I think that's totally irritating. Why can I delete comments from someone who is a stranger?" |  |  |  |
|  | 6.2.3 Navigation | - Notes regarding navigation through the app | "(...) that means you need back and forward options, that you can go to the page you had before and practically always tap back manually without having to think about” |  |  |  |
| 6.3 Lack of clarity |  | - Intuitive use of app content | "(...) I haven't checked that for ages, that I can't click directly on this, that I can't click on this whole field and then I can call up the group, but I have to click exactly on this heading. (...)" |  |  |  |
| 6.4. Proposal of optimization |  | - Suggestions for improvement or ideas regarding the functionality of the app | ---------- |  |  |  |
|  | 6.4.1. Menu | - Feedback on the arrangement of the control panel | "(...) So I would put “exposition” more to the left, huh? (...) I think users find it easier to read from left to right, just as you read, ne? (...)" |  |  |  |
|  | 6.4.2. Module name | - Module names are not intuitively understandable | "(...) I'm struggling a bit with the name "tutorial" (...), because until now I've had it on the screen that a tutorial is more like a manual and these are more like, I'd call it FAQs or something like that." |  |  |  |
|  | 6.4.3. Group content | - Descriptions and contents of the groups should be visible before joining the group | "(...) One should be able to read the description of the particular group before joining it (...)" |  |  |  |
|  | 6.4.4. Optical representation | - The visual design of the app functions - visibility - responsivity | "(...), but somehow, I just don't see such a summary, (...) such a (...) difference or "It wasn't so bad" (...)" |  |  |  |
|  | 6.4.5. Guidance | - introduction to the operation of the app | "I think it's more about using, (...) "You click on this and this button, please, and then this and this happens"(...)" |  |  |  |
|  | |  | 6.4.6. Visibility and Privacy | - View the profiles of other users and see their activities. | "(…) Of course it's a sensitive topic, because I, uh, might be very open about it, but someone else might prefer to keep it private. But you already have such functions in [the app] anyway: Do I want to share this with someone or not, yes? (...)" |  |
|  |  |  | 6.4.7. Information flow | - Reminder, Tutorials, step-by-step text release visualization, diary | "(...) When I'm feeling bad (...), I can immediately display what I've already accomplished or what's good for me at that moment (...)." |  |
